# Computer-aided diagnosis of renal obstruction: utility of log-linear modeling versus standard ROC and kappa analysis

**DOI:** 10.1186/2191-219X-1-5

**Published:** 2011-06-20

**Authors:** Amita K Manatunga, José Nilo G Binongo, Andrew T Taylor

**Affiliations:** 1 Department of Biostatistics and Bioinformatics, Emory University School of Public Health, 1364 Clifton Road NE, Atlanta, GA 30322, USA; 2 Department of Radiology and Imaging Sciences, Emory University School of Medicine, 1364 Clifton Road NE, Atlanta, GA 30322, USA

**Keywords:** Log-linear modeling, Renal obstruction, Diuresis renography

## Abstract

**Background:**

The accuracy of computer-aided diagnosis (CAD) software is best evaluated by comparison to a gold standard which represents the true status of disease. In many settings, however, knowledge of the true status of disease is not possible and accuracy is evaluated against the interpretations of an expert panel. Common statistical approaches to evaluate accuracy include receiver operating characteristic (ROC) and kappa analysis but both of these methods have significant limitations and cannot answer the question of equivalence: Is the CAD performance equivalent to that of an expert? The goal of this study is to show the strength of log-linear analysis over standard ROC and kappa statistics in evaluating the accuracy of computer-aided diagnosis of renal obstruction compared to the diagnosis provided by expert readers.

**Methods:**

Log-linear modeling was utilized to analyze a previously published database that used ROC and kappa statistics to compare diuresis renography scan interpretations (non-obstructed, equivocal, or obstructed) generated by a renal expert system (RENEX) in 185 kidneys (95 patients) with the independent and consensus scan interpretations of three experts who were blinded to clinical information and prospectively and independently graded each kidney as obstructed, equivocal, or non-obstructed.

**Results:**

Log-linear modeling showed that RENEX and the expert consensus had beyond-chance agreement in both non-obstructed and obstructed readings (both *p *< 0.0001). Moreover, pairwise agreement between experts and pairwise agreement between each expert and RENEX were not significantly different (*p *= 0.41, 0.95, 0.81 for the non-obstructed, equivocal, and obstructed categories, respectively). Similarly, the three-way agreement of the three experts and three-way agreement of two experts and RENEX was not significantly different for non-obstructed (*p *= 0.79) and obstructed (*p *= 0.49) categories.

**Conclusion:**

Log-linear modeling showed that RENEX was equivalent to any expert in rating kidneys, particularly in the obstructed and non-obstructed categories. This conclusion, which could not be derived from the original ROC and kappa analysis, emphasizes and illustrates the role and importance of log-linear modeling in the absence of a gold standard. The log-linear analysis also provides additional evidence that RENEX has the potential to assist in the interpretation of diuresis renography studies.

## Background

The increase in the number and complexity of diagnostic studies, subjectivity in image interpretation, physician time constraints, and high error rates have stimulated the development of computer-aided diagnostic (CAD) tools to help nuclear medicine physicians and radiologists interpret studies at faster rate and with higher accuracy [1-5]. The introduction of new decision support tools, however, has raised a critical question: What is the best way to evaluate the performance of these new diagnostic tools? Ideally, the accuracy of a new diagnostic tool should be measured against a gold standard which represents the true status of the disease, i.e., disease present or disease absent. Unfortunately, in many circumstances, a gold standard is not available due to the fact that the gold standard is unacceptably invasive, prohibitively expensive, or simply non-existent [6-9]. A common approach to this problem is to compare the diagnosis of a new CAD tool with those of expert readers. However, since experts do not always agree, the CAD diagnosis is often compared to a consensus diagnosis of experts. The best standard, however, is not how well the new diagnostic tool performs compared to a consensus interpretation of experts but to determine if its performance is *equivalent *to the diagnostic performance of any expert. When the performance of the new CAD tool is equivalent to any expert, the new computer-aided tool can be considered to be sufficient to assist in scan interpretation.

Receiver operating characteristic (ROC) and kappa methodologies have been and continue to be popular methods to assess the reliability of computer-aided diagnosis tools [7,8,10-12], but both of these common approaches have significant limitations. ROC analysis requires an independent measure of truth, and it requires the measure to be dichotomized (e.g., disease present or absent). In practice, image interpretation may not be definitive and the report may be qualified by terms like "indeterminate," "possible" or "questionable." In contrast, kappa statistics [13,14] measure the degree of agreement beyond that expected by chance alone. For example, when there are three categories such as "normal", "equivocal", and "obstruction" in rating of kidney images, the kappa statistic provides a number between 0 and 1, indicating the strength of agreement beyond chance across all categories. A major disadvantage of kappa is that, by construction, it provides an overall summary of beyond-chance agreement across all categories and there is a loss of information [14] in summarizing the data and it does not specifically address how two raters agree on a certain category. Moreover, kappa-type statistics [9,15,16] can be heavily influenced by the distribution of disease in the population as well as by differences or similarities among raters [17]. It is also difficult to interpret the magnitude of the kappa statistic, particularly the degree to which a change can be considered to be an improvement. For example, is kappa = 0.7 clinically superior to kappa = 0.6 in terms of agreement? In fact, common statistical methods such as kappa and ROC are not designed to determine if a computer-aided diagnosis tool provides interpretations that are "equivalent" to expert interpretations and a new framework is needed for addressing these questions.

In this manuscript, we present a statistical modeling approach [16] called log-linear modeling which is more informative and useful for evaluating a new computer-aided diagnostic tool against experts than ROC curves and kappa statistics. This approach can fully characterize the accuracy of computer-aided diagnosis tool against experts by evaluating the pattern of agreement across rating categories. Moreover, it can quantify the magnitude of the agreement and assess its statistical significance. In particular, the modeling approach can address the critical question: Is the performance of a new diagnostic tool equivalent to performance of an expert? To illustrate the added value of log-linear analysis over ROC and kappa, we compared the accuracy of a new CAD approach for the diagnosis of renal obstruction (RENEX) to the diagnosis provided by three experts where RENEX and the experts rated each kidney in a series of diuresis renography studies as non-obstructed, equivocal, and obstructed.

## Methods

Institutional review board approval was obtained for this HIPAA-compliant study; the requirement for informed patient consent was waived. RENEX is a renal expert system for detecting renal obstruction using pre- and post-furosemide Tc-99 m mercaptoacetyltriglycine (MAG3) renal scans [18]. RENEX consists of: (1) a *parameter knowledge library *with the list of the boundary conditions necessary for transforming the values of each quantitative parameter such as time to peak height of the renogram curve or time to half maximum counts (*T*1/2) to a certainty factor describing the degree of abnormality or normality of that parameter, (2) a *knowledge base *of heuristic rules that uses certainty factors describing the degree of normality or abnormality of specific parameters to generate new certainty factors specifying the likelihood of obstruction,(3) and an *inference engine *to combine the certainty factors of the rules and parameters to reach a final certainty factor (conclusion) in regard to obstruction [19]. Detailed description of the architecture of RENEX is presented in a separate publication [18]. RENEX was optimized using pilot data [18] and prospectively validated [10].

This study analyzed a previously published data base that compared diuresis renography scan interpretation generated by RENEX with the consensus and individual scan interpretation of three experts using ROC and kappa analysis [10]. The database consisted of 95 patient studies (55 males and 40 females with a mean age of 58.6 years, SD = 16.5) and contained 185 kidneys classified by RENEX and three experts as obstructed, equivocal, or non-obstructed. Readers were defined as "expert" on the basis of the fact that each reader had > 20-years experience in full-time academic nuclear medicine, had multiple publications in renal nuclear medicine and have been invited to give renal nuclear medicine educational session as national radiology and nuclear medicine meetings. The experts were blinded to clinical information and had prospectively and independently rated each kidney as obstructed, equivocal, or non-obstructed; a consensus reading was subsequently obtained by resolving the differences of expert readings. RENEX analyzed the 95 patient studies based on quantitative parameters automatically extracted from baseline and furosemide acquisitions [20,21] and used clinically validated optimal cut-off points to classify a kidney as obstructed, equivocal, or non-obstructed.

The diuresis renography protocol was a two-stage acquisition based on a minor modification of the consensus recommendations [22]. A 24-min baseline Tc-99 m MAG3 scan was first obtained. If there was prompt bilateral drainage, obstruction was excluded and furosemide was not administered; if there was delayed drainage in one or both kidneys, the patient received furosemide and an additional 20-min scan was obtained. Exclusion of clearly non-obstructed patients (those with a normal baseline acquisition who, consequently, did not receive furosemide) weighted the study population toward a higher percentage of patients with an indeterminate or obstructed kidney.

### Statistical modeling

Our primary interest in using a log-linear modeling approach was to characterize the overall structure of agreement present in the data. Carefully considering possible reasons why agreement is present, our modeling procedure can quantify the pattern and magnitude of agreement. For example, we can address the question as to whether agreement in the data is due to chance or due to actual agreement among the raters. Moreover, the actual rater agreement can be further divided into category-specific agreement components (obstructed, equivocal, non-obstructed) because it is possible to have different agreement patterns in each of the different response categories. These various components were incorporated by specifying a series of statistical models, starting with the independence model. Goodness-of-fit tests were conducted to select the best model that characterizes the structure of agreement present in the data. For model selection, significance level *α *= 0.05 was used. Once a model was selected, we used the more conservative significance level of *α *= 0.01 due to multiple comparisons.

We employed an appropriate log-linear model to address the two questions:

1. How does the rating of RENEX compare with the consensus interpretation of three experts?

2. How does the rating of RENEX compare with the interpretations of the individual experts?

To address the first question, we treated consensus reading as the interpretation of one expert. To address the second question, we evaluated two agreement patterns: (1) pairwise agreement (i.e., two raters) and (2) three-way agreement (i.e., three raters). This evaluation allowed us to determine if the performance of RENEX was equivalent to the performance of expert readers.

### Comparison of RENEX to the consensus interpretation

To investigate the agreement between RENEX and expert consensus, we developed a sequence of nested log-linear models starting with the simplest model, a baseline model for Table 1. This model is also called the independence model which assumes that the agreement between consensus and RENEX is due to chance alone. The cell count in the 3 × 3 table is modeled via two components: a separate effect due to RENEX and a separate effect due to consensus interpretation.

**Table 1:** Number of kidneys rated by RENEX and the consensus readings of experts (*n *= 185)

RENEX reading	Consensus reading		
	Non-obstructed	Equivocal	Obstructed
Non-obstructed	101	7	1
Equivocal	14	13	2
Obstructed	5	9	33

Experts are expected to agree among themselves more often than chance would allow. In this case, expert ratings will not be independent and an association in the 3 × 3 contingency table will exist. The resulting rating pattern may thus be described by a configuration with a larger number of counts on the main diagonal than would be expected under independence. If this pattern occurs, the independence model fits the data poorly. When the independence model is not adequate to explain the data, a component measuring the extra agreement present on the main diagonal is added to the model. This model is referred to as the homogeneous agreement model and assigns equal strength of agreement between RENEX and consensus readings across each category (non-obstructed, equivocal, and obstructed). The homogeneous agreement model thus has two components: the first representing chance, the second representing agreement. A significant positive agreement of the second component suggests positive agreement beyond that expected by chance.

When the homogeneous agreement model still cannot adequately capture the agreement information in the data, the homogeneous agreement term is replaced by three terms representing different agreement strengths in each reading category. This is called the non-homogeneous agreement model. Note that if all the categories have a uniform level of agreement, we will have the homogeneous agreement model. The modeling procedure is described in detail in the appendix.

The independence model, homogeneous agreement model, and non-homogeneous agreement model form a nested sequence of models. As such, a likelihood-ratio test can used to examine the improvement in fit. Regression coefficients associated with the agreement terms are calculated under the best-fitting model.

### Comparison of RENEX to expert raters

The goal of this comparison is to determine if the performance of the RENEX is actually equivalent to that of expert readers. There were three experts and RENEX; hence, the data could be considered as having four raters, each evaluating kidneys into three categories. To address the question of equivalence of RENEX with respect to individual expert readers, we compared the agreement within experts to the agreement between RENEX and experts. Because we had three experts, it was natural to consider two agreement patterns: (1) pairwise agreement and (2) three-way agreement. We thus examined the agreement between RENEX and individual experts by taking two or three raters at a time.

As before, we first started with the independence model which included effects due to all three experts and RENEX. Next, a model allowing pairwise agreement was considered. This was done by adding terms to the previous model which are effects due to pairwise agreement among experts and effects due to pairwise agreement between RENEX and an expert. This is the homogenous pairwise agreement model. The third model extended this homogeneous model by expanding the terms described in the homogeneous pairwise agreement model to reflect different strengths of pairwise agreement according to response categories. Finally, we considered the three-way agreement model by including effect due to three-way agreement among experts and three-way agreement among expert and RENEX. The modeling procedure is detailed in the appendix.

## Results

### Agreement between RENEX and the Consensus Interpretations

The agreement between RENEX and the consensus readings for 185 kidneys is shown in Table 1. The expert system agreed with the consensus reading in 84% (101/120) of non-obstructed kidneys, in 92% (33/36) of obstructed kidneys, and in 45% (13/29) of equivocal kidneys.

To determine the best model for the agreement between RENEX and expert consensus, a series of models were examined. The likelihood-ratio statistics (*G*^2^) for both the independence model, *G*^2 ^= 138.55 [*df *(degrees of freedom) = 4, *p *< 0.001] and homogeneous agreement model *G*^2 ^= 21.38 (*df *= 3, *p *< 0.001) indicated that neither of these models was adequate to describe the data. (When performing the likelihood-ratio test in log-linear analysis, a model is considered adequate if its *p *value is *at or above 0.05*). For the non-homogeneous agreement model, *G*^2 ^= 0.16 (*df *= 1, *p *= 0.69) showing the adequacy of the model in describing the pattern of agreement; the agreement pattern in the data favors assigning different strengths of agreement to the three response categories.

The results based on the non-homogeneous agreement model are displayed in Table 2. Of the 185 kidneys, expert consensus classified 65%, 16%, and 19%, respectively, as non-obstructed, equivocal, and obstructed. On the other hand, RENEX classified 59%, 16%, and 25%, respectively, as non-obstructed, equivocal, and obstructed. The regression coefficients for the non-obstructed, equivocal, and obstructed categories were, respectively, 1.57 (*p *< 0.0001), -0.28 (*p *= 0.37), and 1.82 (*p *< 0.0001). These coefficients show that there was a significant positive agreement between RENEX and expert consensus in the non-obstructed and obstructed categories.

**Table 2:** Agreement between RENEX and consensus for each rating category

Category	Regression coefficient, δ^a ^(SE)	*p *value*
Non-obstructed (*δ*_1_)	1.57 (0.30)	< 0.0001
Equivocal (*δ*_2_)	-0.28 (0.31)	0.37
Obstructed (*δ*_3_)	1.82 (0.36)	< 0.0001

* *p *< 0.05 indicates there is a significant beyond-chance agreement between RENEX and consensus readings in the corresponding category. ^a^The *δ*s reflect the strength of the beyond-chance agreement within each specific category.

Both kappa [10] and log-linear analysis showed that consensus and RENEX interpretations agreed beyond chance; however the log-linear modeling approach further suggested that the agreement pattern among the three response categories was not uniform. In particular, RENEX and expert consensus rated the renal scans with high agreement in the non-obstructed and obstructed categories while they did not seem to agree well in the equivocal category.

### Agreement between RENEX and the Individual Experts

To address the question of whether RENEX is equivalent to an expert, we examined the pattern of agreement between experts and RENEX by considering pairwise agreement and three-way agreement.

1. Pairwise agreement within experts and between experts and RENEX. Based on likelihood-ratio tests, the non-homogeneous model (*G*^2 ^= 58.69, *df *= 54, *p *= 0.31) was preferred over the independence model (*G*^2 ^= 530.70, *df *= 72, *p *< 0.0001) and the homogeneous model (*G*^2 ^= 102.61, *df *= 66, *p *< 0.01). The results based on the non-homogeneous agreement model are displayed in Table 3. Although the pattern of pairwise agreement is not apparent across all raters, coefficients in the non-obstructed category seem to indicate positive significant agreement. A hypothesis-testing approach provides more insight into the pattern of agreement, which is described next.

**Table 3:** Pairwise agreement within experts and between experts and RENEX

	Log-linear model coefficients					
	Between experts			RENEX and expert		
	*E*_1_ *E*_2_	*E*_1_ *E*_3_	*E*_2_ *E*_3_	*R E*_1_	*R E*_2_	*R E*_3_
	[*δ*_m1_, (SE)]	[*δ*_*m*2_, (SE)]	[*δ*_*m*3_, (SE)]	[*θ*_*m*1_, (SE)]	[*θ*_*m*2_, (SE)]	[*θ*_*m*3_, (SE)]
Non-obstructed	1.58* (0.42)	0.36 (0.40)	0.91 (0.46)	0.26 (0.40)	1.08* (0.41)	0.84* (0.32)
Equivocal	-0.12 (0.40)	0.89 (0.37)	-0.58 (0.44)	-0.06 (0.37)	-0.28 (0.39)	0.47 (0.33)
Obstructed	0.78 (0.46)	-0.07 (0.43)	1.47* (0.45)	0.48 (0.43)	1.08 (0.44)	0.41 (0.38)
	*p *values for tests of hypothesis					
	Among experts		RENEX and expert		Experts vs. RENEX	
	*H*_0_: *δ*_*m*1_= *δ*_*m*2_= *δ*_*m*3_^a^		*H*_0_: *θ*_*m*1_= *θ*_*m*2_= *θ*_*m*3_^b^		*H*_0_: *δ*_*m*1 _+ *δ*_*m*2 _+ *δ*_*m*3 _= *θ*_*m*1 _+ *θ*_*m*2 _+ *θ*_*m*3 _^c^	
Non-obstructed	0.15		0.47		0.41	
Equivocal	0.08		0.34		0.95	
Obstructed	0.11		0.58		0.81	

* Significant positive pairwise agreement at *α *= 0.01. ^a^Hypothesis that the overall pairwise agreement among experts is the same. The *δ*s reflect the strength of the beyond-chance agreement between two raters within each specific category. ^b^Hypothesis that the overall pairwise agreement between RENEX and each expert is the same. The *θ*s reflect the strength of the beyond-chance agreement between RENEX and an expert within each specific category. ^c^Hypothesis that the overall pairwise agreement among experts is the same as the overall pairwise agreement between each expert and RENEX.

Tests of hypothesis in Table 3 indicated that for all response categories, the pairwise agreement among experts was the same (*p *= 0.15, 0.08, 0.11, respectively, for the non-obstructed, equivocal, and obstructed categories). Moreover, the pairwise agreement between RENEX and an expert are also the same (*p *= 0.47, 0.34, 0.58, respectively, for the non-obstructed, equivocal and obstructed categories). Finally, the overall pairwise agreement between two experts and the overall pairwise agreement between RENEX and an expert are the same (*p *= 0.41, 0.95, 0.81, respectively). Hence, pairwise agreement appeared to remain the same when an expert was replaced by RENEX. In terms of pairwise comparisons, the performance of RENEX is equivalent to that of an expert.

2. Three-way agreement within experts and between experts and RENEX. Based on likelihood-ratio tests, the non-homogeneous agreement model (*G*^2 ^= 61.63, *df *= 60, *p *= 0.42) was preferred over the homogeneous agreement model (*G*^2 ^= 113.60, *df *= 68, *p *< 0.001) showing that the pattern of three-way agreement in the data is different for the three response categories. The results based on the non-homogeneous agreement model are displayed in Table 4. Coefficients in the non-obstructed and obstructed categories indicate significant positive agreement. Tests of hypothesis suggest that agreement among three experts is the same as agreement among two experts and RENEX for the non-obstructed (*p *= 0.79), obstructed (*p *= 0.49) and equivocal categories (*p *= 0.03). Since none of these values reached the level of significance (*p *≤ 0.01, Table 4), RENEX appears to be equivalent to an expert in all three categories.

**Table 4:** Three-way agreement within experts and between experts and RENEX

	Log-linear model coefficients			
Response category	Experts	RENEX and experts		
	*E*_1_ *E*_2_ *E*_3_	*E*_1_ *E*_2_ *R*	*E*_1_ *E*_3_ *R*	*E*_2_ *E*_3_ *R*
	[*δ_m_*, (SE)]	[*δ*_*m*1_, (SE)]	[*δ*_*m*2_, (SE)]	[*δ*_*m*3_, (SE)]
Non-obstructed	0.82* (0.29)	0.98* (0.30)	0.60 (0.38)	0.60 (0.38)
Equivocal	0.64 (0.28)	-0.34 (0.37)	0.32 (0.28)	-0.21 (0.34)
Obstructed	0.08 (0.97)	1.48* (0.42)	-0.45 (0.98)	1.89* (0.35)
		*p *values for tests of hypothesis		
		*H*_0_: *δ*_*m *_= 1/3 [*θ*_*m*1 _+ *θ*_*m*2 _+ *θ*_*m*3_]^a^		
Non-obstructed		0.79		
Equivocal		0.03		
Obstructed		0.49		

* Significant positive three-way agreement at *α *= 0.01. ^a^Tests the hypothesis that the overall three-way agreement among experts is the same as the overall three-way agreement between RENEX and any two experts

## Discussion

One goal of this manuscript was to show the advantages of log-linear regression analysis when a gold standard is absent by analyzing a previously published database that assessed the accuracy of computer-aided diagnosis of renal obstruction against the diagnosis provided by expert readers using kappa and ROC methods [10]. In the ROC analysis, the expert consensus was used as the gold standard but this approach is problematic because ROC analysis should have a gold standard independent of the test under evaluation. Unfortunately, this problem occurs whenever an expert panel is used as the gold standard. Secondly, ROC analysis requires just two categories, disease present or disease absent. To apply ROC analysis, equivocal interpretations have to be placed into the disease present or disease absent category [10]. This requirement may obscure critical information and fails to represent the clinical setting where some interpretations are, in fact, equivocal.

An alternative to ROC analysis is kappa analysis. The weighted kappa statistic between RENEX and expert consensus readings was 0.72 which indicated good agreement between RENEX and experts [10]. The weighted kappa coefficients between each pair of experts and between RENEX and each expert also ranged from 0.61 to 0.73 [10]; this close agreement of kappa coefficients suggested that RENEX was performing similarly to an expert. However, kappa analysis does not provide a framework for evaluating the pattern of agreement across different categories. In our analysis, we found that RENEX has better agreement with consensus and experts in obstructed and non-obstructed categories, but not in the equivocal category.

Log-linear models [16] can establish the general pattern and magnitude of agreement which provides valuable information for improving the reliability of a computer-aided diagnosis system. In a log-linear framework, agreement is specified by two components: one represents the effect of chance and the other represents the effect of rater agreement beyond chance. Compared to a summary statistic like kappa, log-linear models provide a straightforward test of the magnitude of the difference in agreement and also provide more information about agreement such as the structure and pattern of the agreement across categories.

For example, a kappa of 0.72 only gives us a sense that RENEX agrees well with consensus, but it is hard to say whether the agreement is high in all three reading categories (obstructed, equivocal or non-obstructed) or exists only in some categories. Log-linear modeling shows us that the overall agreement between RENEX and consensus across the reading categories exists beyond chance (*p *< 0.001). Furthermore, the significance tests under a non-homogeneous agreement model suggest that the beyond-chance agreement mainly comes from the ratings in non-obstructed and obstructed categories. To determine if a new diagnostic tool is "equivalent to" an expert, the advantage of log-linear models becomes more apparent since the agreement among various combinations of raters can be specified in one single model and can be tested directly in this context (Table 4). The results of significance tests led us to conclude that RENEX behaves equally to an expert in all reading categories.

## Conclusions

Log-linear modeling (1) provided more insight into the pattern and magnitude of inter-rater agreement than ROC and kappa analysis, (2) showed that RENEX performed as well as any expert reader particularly rating in obstructed and non-obstructed categories, and (3) should be considered when a gold standard is absent. This analysis provides additional evidence that the renal expert system (RENEX) interprets diuresis renography studies as well as human experts and has the potential to assist in the interpretation of diuresis renography studies.

## Abbreviations

ROC: receiver operating characteristic; CAD: computer-aided diagnosis; df: degrees of freedom; SD: standard deviation.

## Competing interests

ATT receives royalties from the sale of the application software QuantEM™ related to the research described in this article. The terms of this arrangement have been reviewed and approved by Emory University in accordance with its conflict-of-interest practice. ATT used to consult for Mallinckrodt, Inc (now Covidien); he has not consulted for the past 3 years.

## Authors' contributions

AM and JNGB designed the log-linear model, conducted the statistical analysis, wrote the appendix, and assisted in writing the manuscript. ATT designed the project, wrote the first draft of the manuscript, critically reviewed the data and completed the final version of the manuscript following input from AM and JNGB.

## Authors' information

AM is Professor of Biostatistics and Bioinformatics, Rollins School of Public Health, Emory University; JNGB is Research Associate Professor in the Department of Biostatistics and Bioinformatics, Rollins School of Public Health, Emory University; ATT is Professor of Radiology, Emory University School of Medicine, Atanta, GA, USA.

## Appendix

### Log-linear modeling

#### Comparison of RENEX vs. consensus

Let *u_ij _*be the expected cell count in a 3 × 3 contingency table where the consensus reading falls in the *i*th category and the RENEX reading falls in the *j*th category (see Table 1). *u *represents the overall effect,  represents the effect due to the *i*th level of consensus, and  represents the effect due to the *j*th level of RENEX. Similar to the regression framework, a series of models is fitted to determine the pattern of agreement. The simplest model to start with is the independence model, which can be written as.

The next model to be considered is the homogeneous agreement model which assumes the same strength of agreement across the three reading categories. That is,

where *I *= 1 if RENEX agrees with CONSENSUS and 0 otherwise. The parameter *δ *indicates the beyond-chance homogeneous agreement. When *δ *is zero, the homogeneous agreement model reduces to the independence model.

The third model to be considered is the non-homogeneous agreement model which assumes a different strength of agreement for different reading categories. That is,

where *I*_1 _= 1 RENEX agrees with CONSENSUS on the first category and 0 otherwise; *I*_2 _= 1 RENEX agrees with CONSENSUS on the second category and 0 otherwise, and so on.

If the strength in agreement for different reading categories is the same, then δ_1_=δ_2_=δ_3_, and the non-homogeneous agreement model reduces to the homogeneous agreement model.

For our data, we fitted these three models using Proc CATMOD in SAS software [23]. For our data, the best model was the non-homogeneous model. Table 4 shows the regression coefficients, standard errors, and *p *values.

#### Comparison of RENEX vs. individual expert readers

In the pairwise homogeneous agreement model, the agreement component assumes the equal strength of agreement between any pair of raters:

The superscripts *E*_1_, *E*_2_, *E*_3_, and *R *refer to the main effect of expert 1, expert 2, expert 3, and RENEX, respectively. *I*_1 _= 1 if experts 1 and 2 agree, 0 otherwise; *I*_2 _= 1 if experts 1 and 3 agree, 0 otherwise; and so on.

When *δ*_1 _= *δ*_2 _= *δ*_3 _= *θ*_1 _= *θ*_2 _= *θ*_3 _= 0, the homogeneous pairwise agreement reduces to the independence model. To determine whether RENEX is behaving similar to experts, we tested the null hypothesis: H_0_: *δ*_1 _+ *δ*_2 _+ *δ*_3 _= *θ*_1 _+ *θ*_2 _+ *θ*_3_.

The next model to be considered was the non-homogeneous pairwise agreement model which permitted a different strength of agreement for different reading categories. That is,

*I*_11 _= 1 if experts 1 and 2 agree on the first category, 0 otherwise; *I*_12 _= 1 if experts 1 and 3 agree on the first category, 0 otherwise; and so on. Here, we were interested in testing the null hypotheses for the mth category: *H*_0_: *δ*_m1 _+ *δ*_m2 _+ *δ*_m3 _= *θ*_m1 _+ *θ*_m2 _+ *θ*_m3_.

The three-way agreement can be modeled in a similar way by appropriately changing the definitions of *δ*s and *θ*s to accommodate three-way agreement.

As before, we fit these models using Proc CATMOD in SAS software [23]. For our data the best models were the non-homogeneous pairwise agreement model and the non-homogeneous three-way agreement model. Table 4 shows the regression coefficients, standard errors, and *p *values.
